# Supplementary material for: Active Compounds in Zingiber officinale as Possible Redox Inhibitors of 5-Lipoxygenase Using an In Silico Approach
Source: Int J Mol Sci. 2022 May 29;23(11):6093. doi: 10.3390/ijms23116093 (PMC9181373; doi:10.3390/ijms23116093)
Supplement: Supplementary file 1 [file ijms-23-06093-s001.zip › ijms-1668787-supplementary.pdf]

Supplementary Table S1. Coupling energies (kcal/mol) between ligands and 5-LOX

Compound	ΔG (Kcal/mol)*
6-gingerol	-5.9
6-shogaol	-6.2

Note: *Binding free energy (Kcal/mol)
